# Supplementary figures and images for: Protein production from HEK293 cell line-derived stable pools with high protein quality and quantity to support discovery research
Source: PLoS One. 2023 Jun 2;18(6):e0285971. doi: 10.1371/journal.pone.0285971 (PMC10237474; doi:10.1371/journal.pone.0285971)

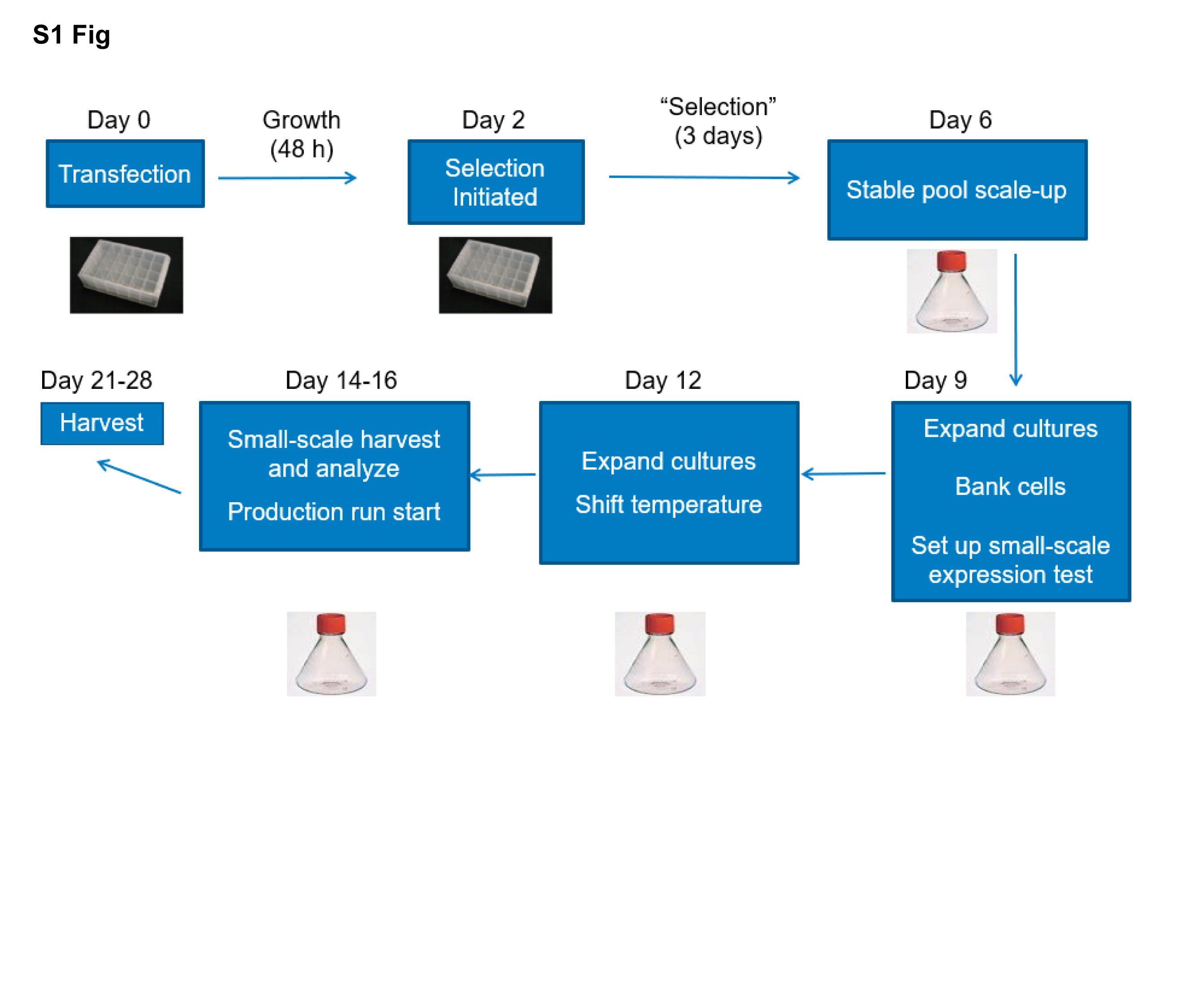

Supplement: S1 Fig — The CHO-K1 stable pool workflow, describing seven steps from co-transfection of PB transposase and PB expression vectors on day 0 to harvest of production cultures (in liters) on day 21-day 28. (TIF) [file pone.0285971.s001.tif]

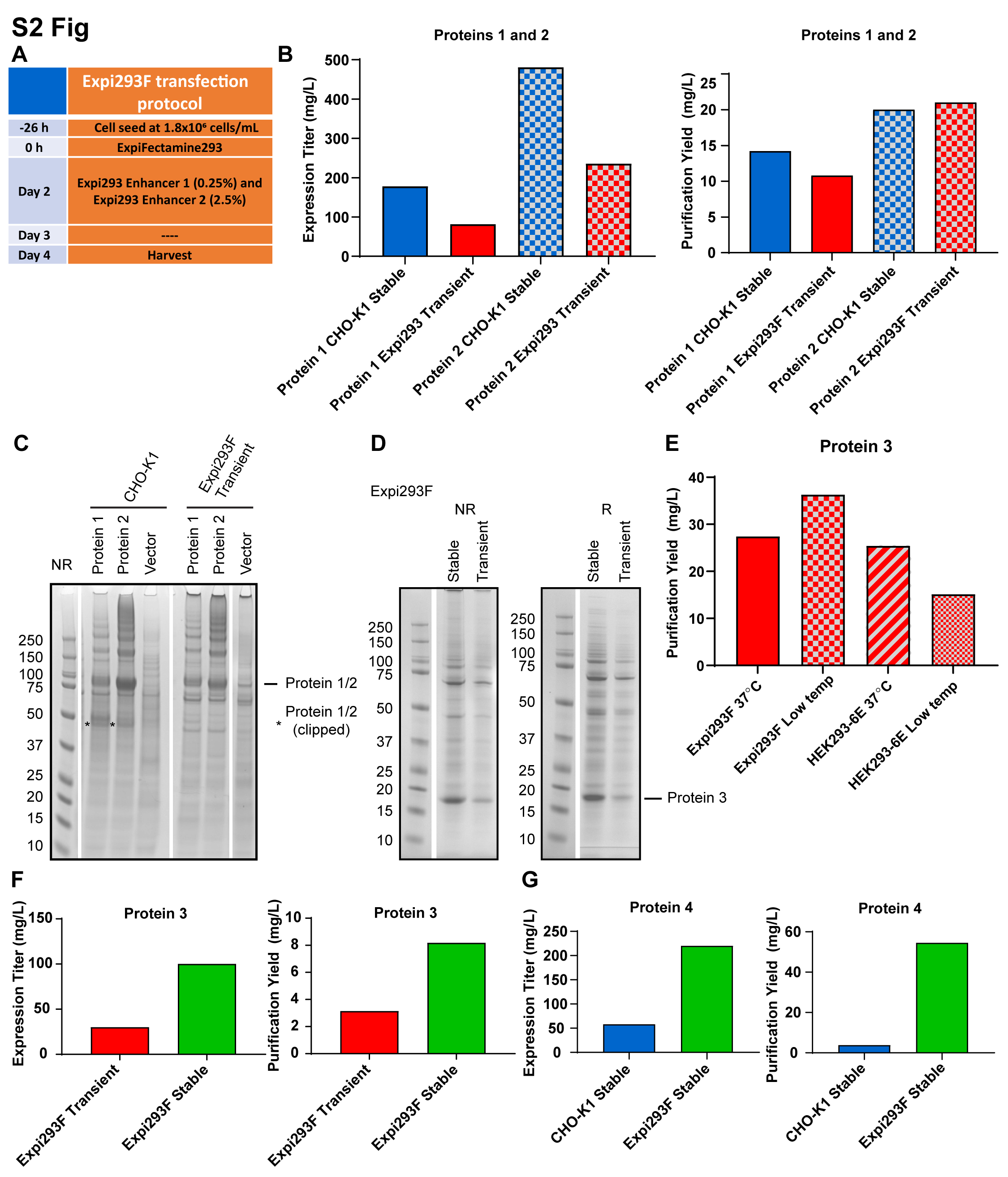

Supplement: S2 Fig — (A) Expi293F transient expression protocol. (B) Expression titer and purification yield of proteins 1 and 2 expressed from CHO-K1 stable pools and Expi293F transient expression are shown. (C) Conditioned media (CMs) from CHO-K1 stable pools and Expi293F transiently expressed proteins 1, 2 or an empty vector and (D) CMs from Expi293F stable pools and transiently expressed protein 3 were analyzed by SDS-PAGE and gels were stained with Coomassie blue. * indicates the clipped fragments of proteins 1 and 2 in CHO-K1 stable pools. (E) Protein 3 transiently expressed from Expi293F and HEK293-6E under different conditions was purified by a one-step metal affinity chromatography. Purification yield is shown. (F) As in (B), but with protein 3 expressed from Expi293F transient and stable pools. (G) As in (B), but with protein 4 expressed from CHO-K1 and Expi293F stable pools. (TIF) [file pone.0285971.s002.tif]

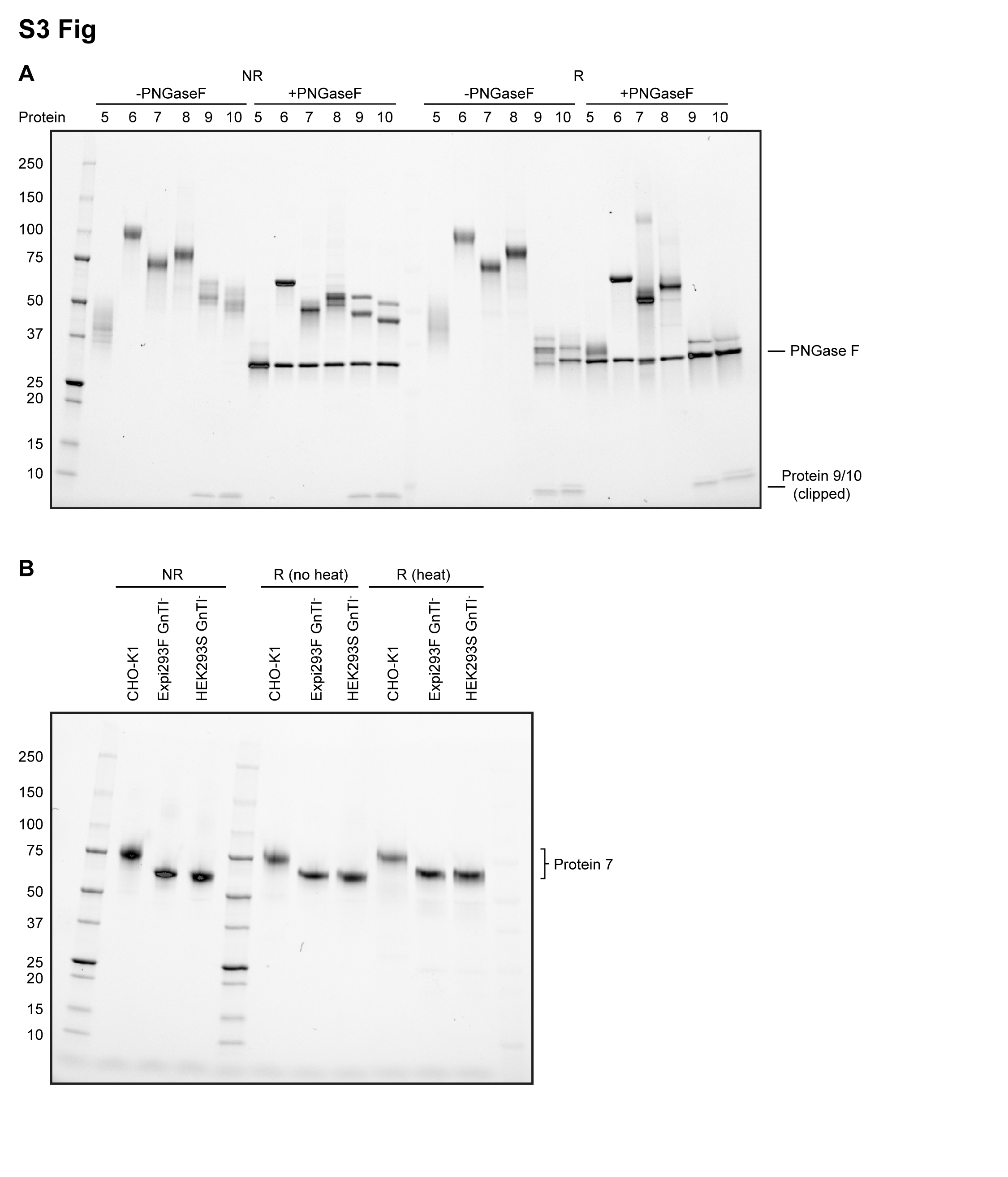

Supplement: S3 Fig — (A) SDS-PAGE analysis of proteins 5–10 purified from CHO-K1 with or without PNGase F digestion under non-reducing and reducing conditions. (B) Protein 7 expressed from CHO-K1, Expi293F GnTI- and HEK293S GnTI- were analyzed by SDS-PAGE under non-reducing and reducing (with or without being heated). Both gels were visualized by stain-free imaging. (TIF) [file pone.0285971.s003.tif]

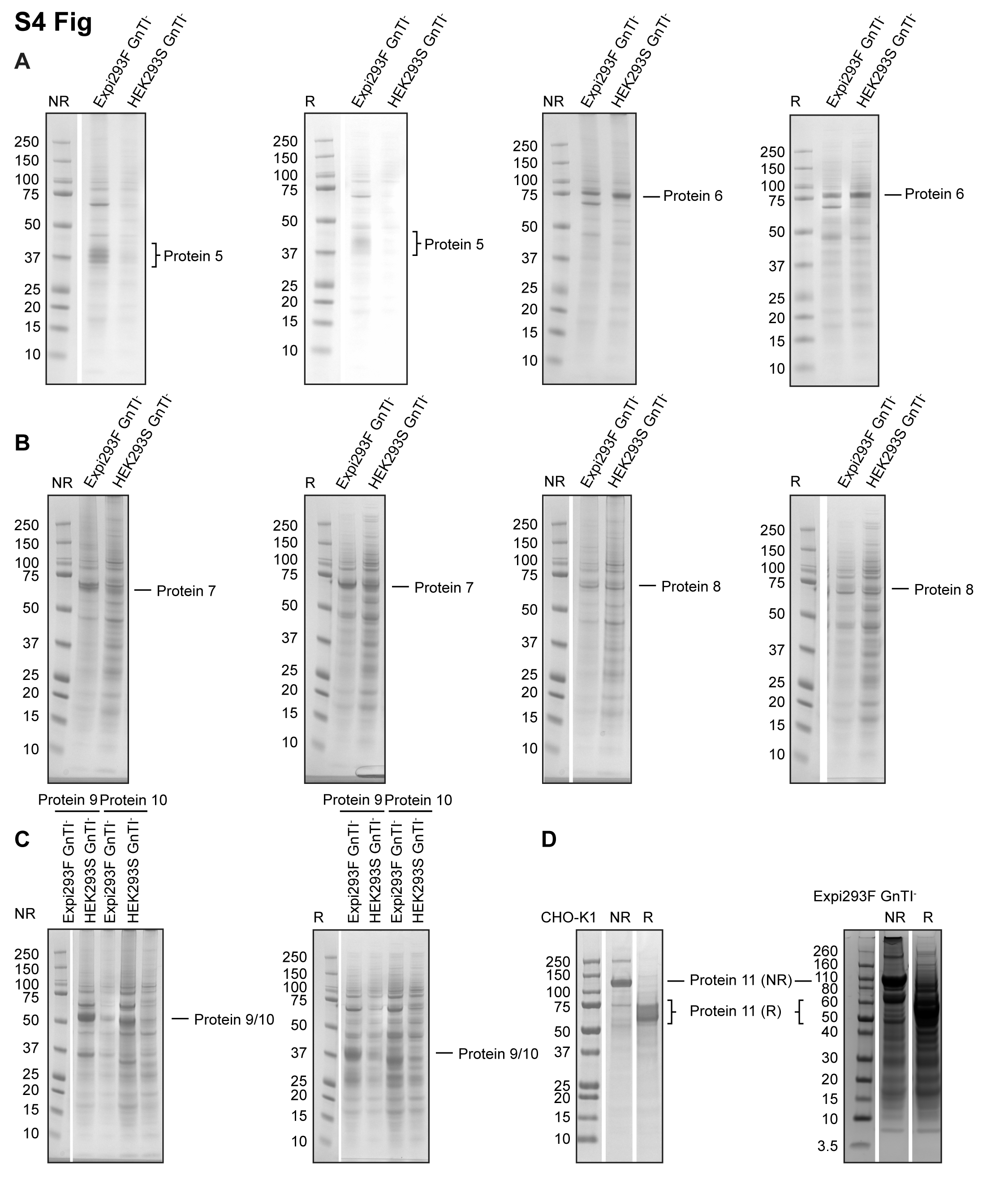

Supplement: S4 Fig — CMs of CHO-K1, Expi293F GnTI- and HEK293S GnTI- expressing (A) proteins 5 and 6, (B) 7 and 8, (C) 9 and 10 and (D) 11 were analyzed by SDS-PAGE under non-reducing and reducing conditions. Gels were stained by Coomassie blue. For protein 11, only CMs of CHO-K1 and Expi293F GnTI- were analyzed. (TIF) [file pone.0285971.s004.tif]

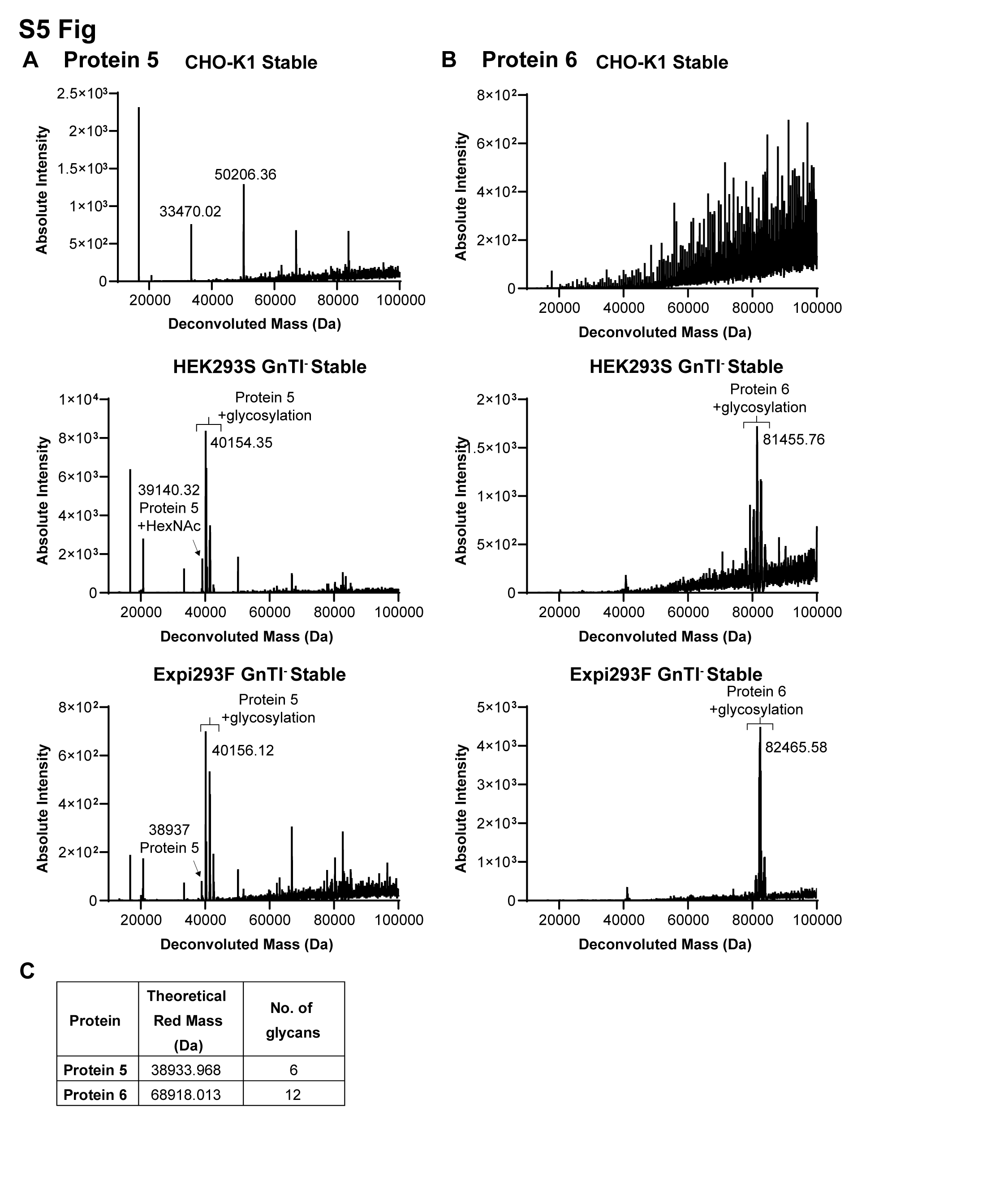

Supplement: S5 Fig — Intact mass analysis suggests that proteins 5 and 6 in GnTI- stable pools are less glycosylated than in CHO-K1 stable pools. (A) Protein 5 and (B) protein 6 purified from CHO-K1, HEK293S GnTI- and Expi293F GnTI- stable pools were subject to intact mass analysis under reducing conditions without deglycosylation. Deconvoluted zero-charge mass spectra are shown. (C) Table showing theoretical reduced molecular masses of proteins 5 and 6 and the number of N-linked glycosylation sites. (TIF) [file pone.0285971.s005.tif]

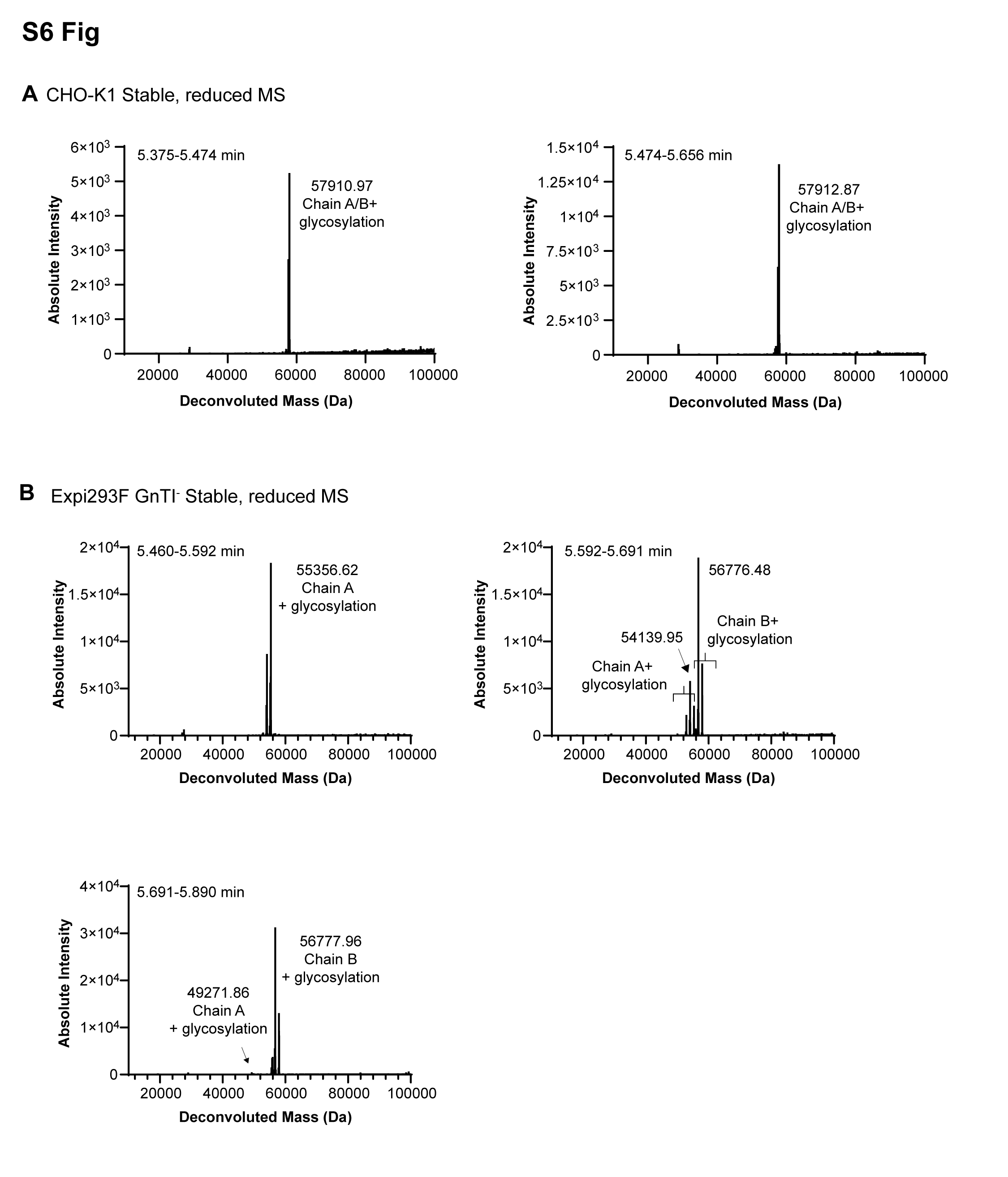

Supplement: S6 Fig — Deconvoluted zero-charge mass spectra of protein 11 expressed from (A) CHO-K1 stable and (B) Expi293F GnTI- stable under reducing conditions without deglycosylation are shown for different retention times. The detected molecular masses of both chains A and B of protein 11 are mostly smaller in Expi293F GnTI- than in CHO-K1. (TIF) [file pone.0285971.s006.tif]
