# Supplementary material for: Protein production from HEK293 cell line-derived stable pools with high protein quality and quantity to support discovery research
Source: PLoS One. 2023 Jun 2;18(6):e0285971. doi: 10.1371/journal.pone.0285971 (PMC10237474; doi:10.1371/journal.pone.0285971)

Fig 1_raw_images

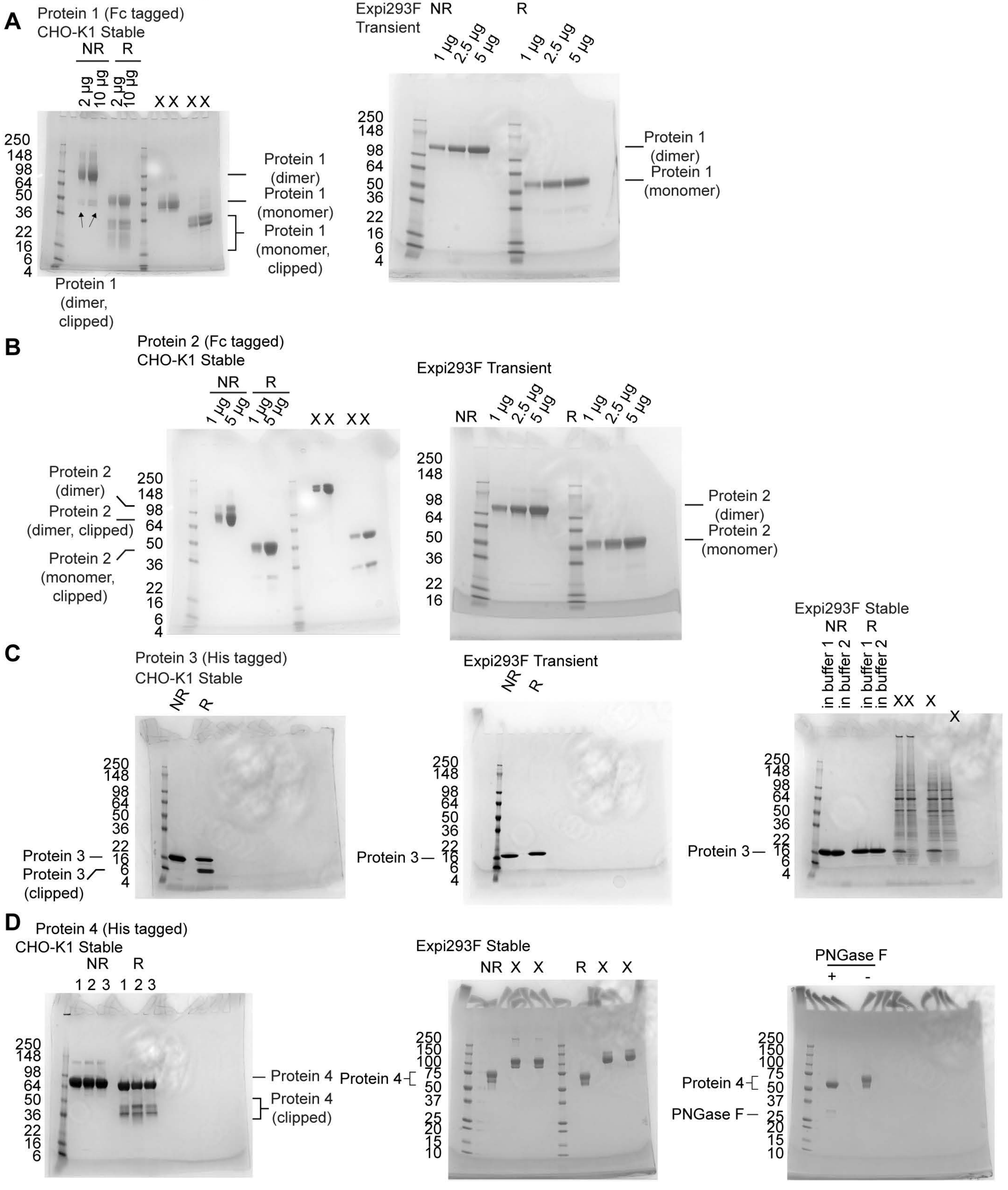

Fig 5_raw_images

A

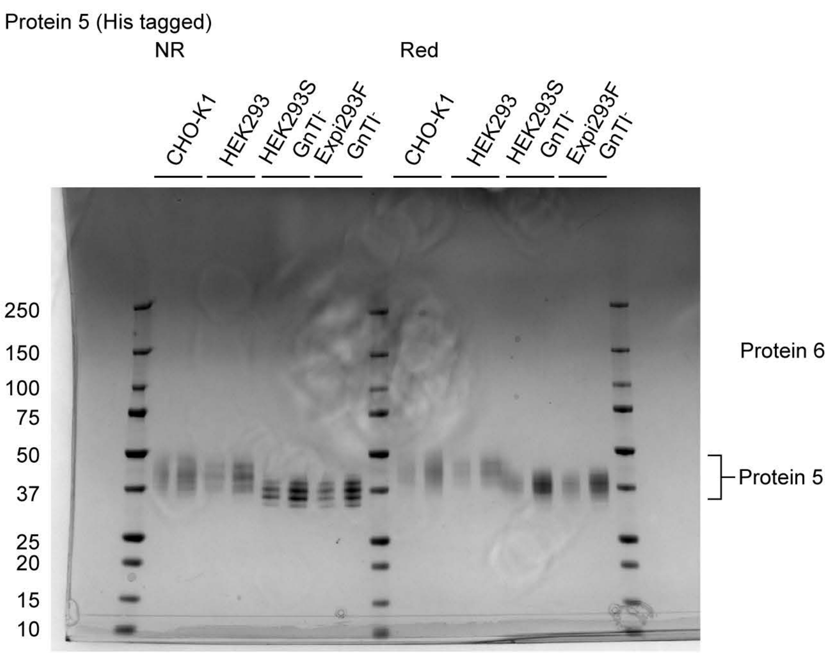

B

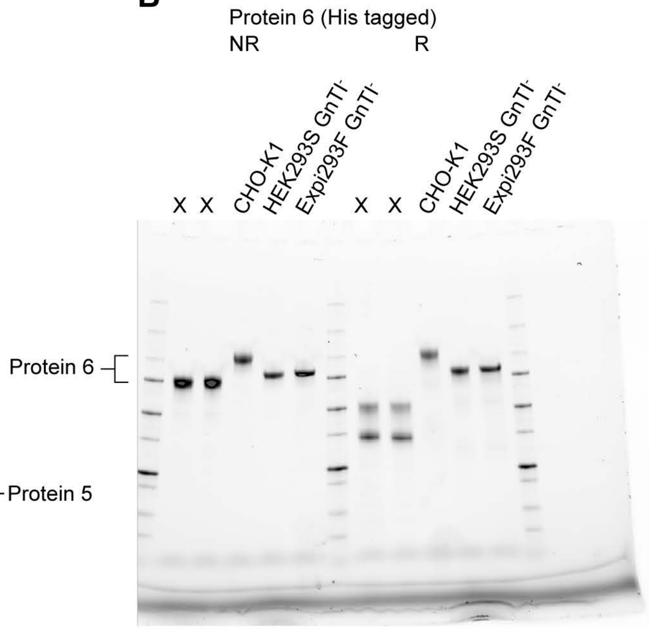

E

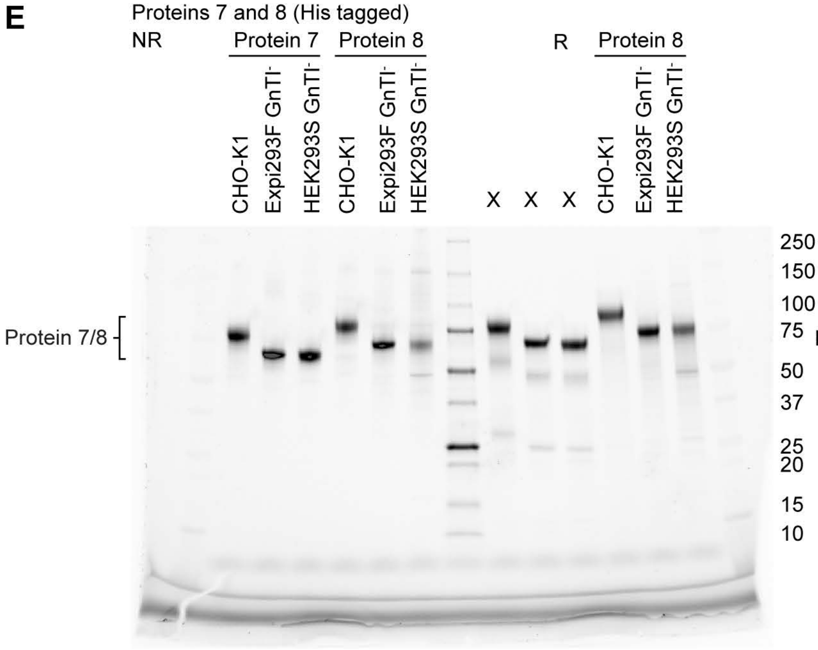

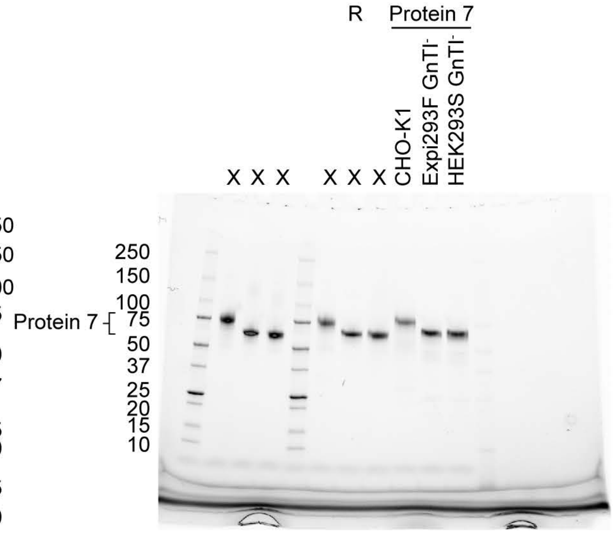

Fig 6_raw_images

A Proteins 9 and 10 (His tagged)

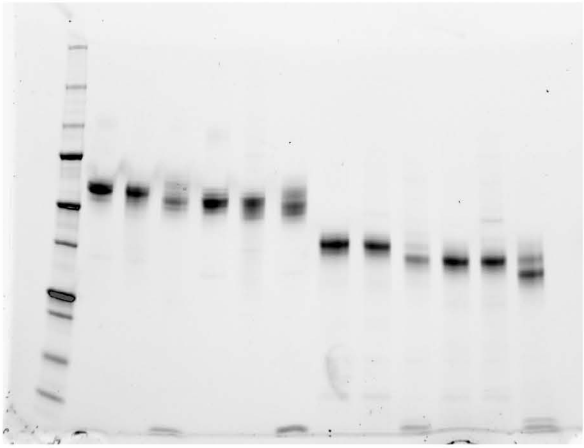

B

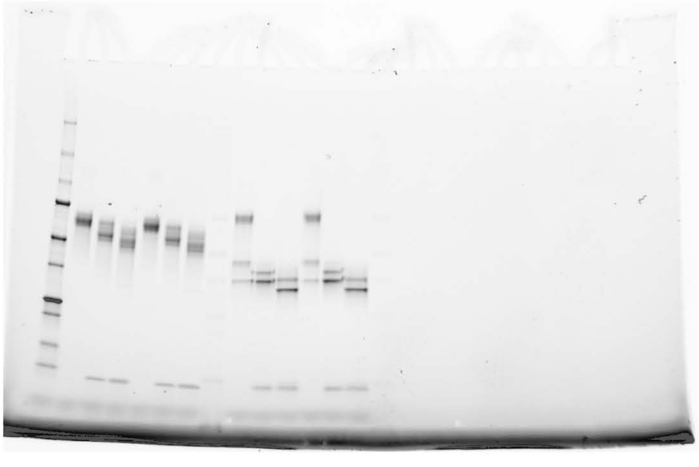

Fig 7_raw_images

B Protein 11 (Fc tagged)

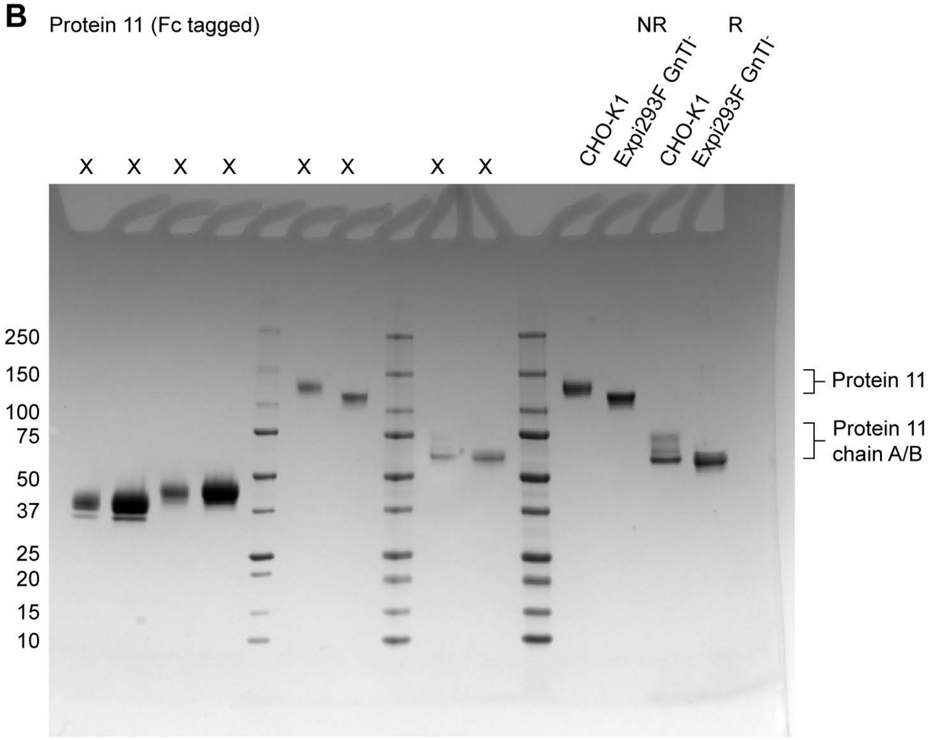

S2_raw_images

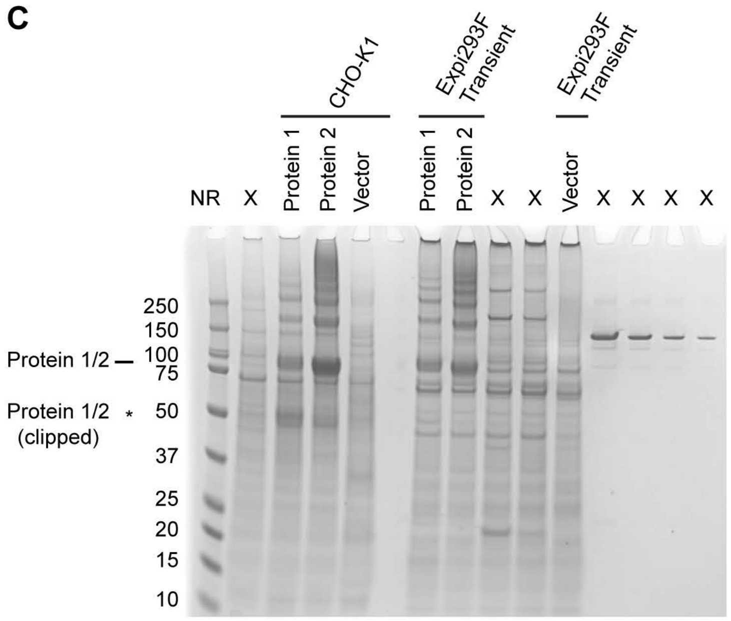

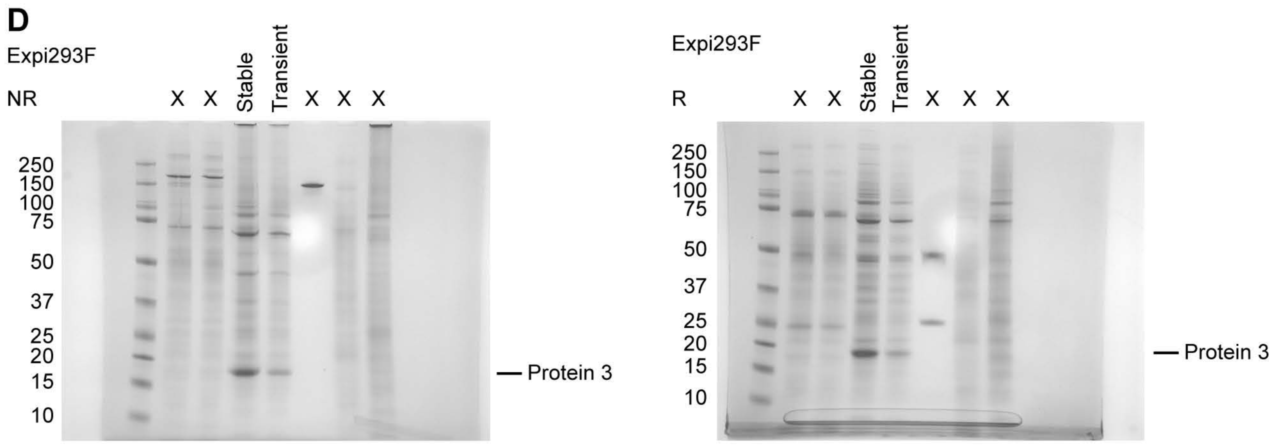

S3_raw_images

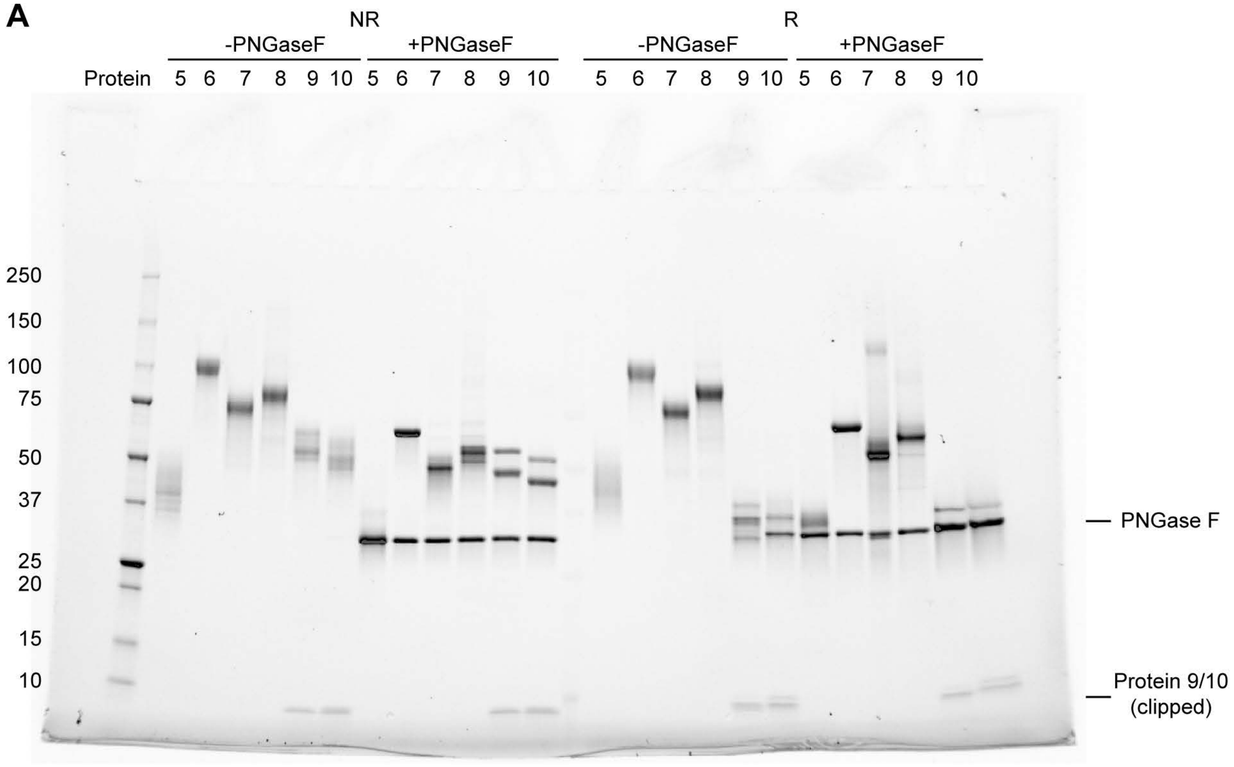

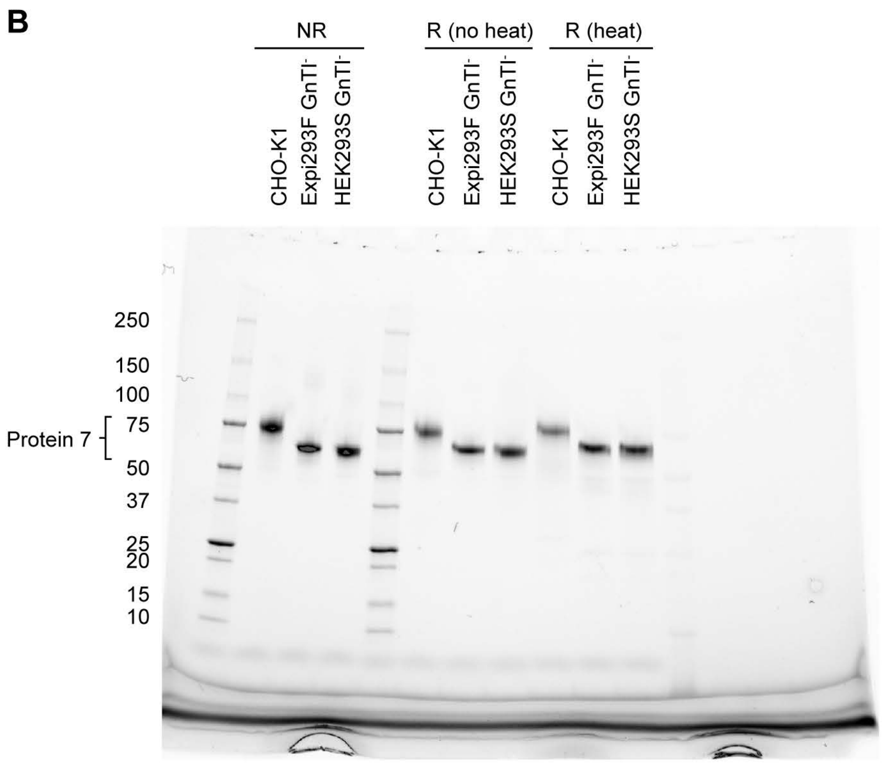

S4_raw_images

A

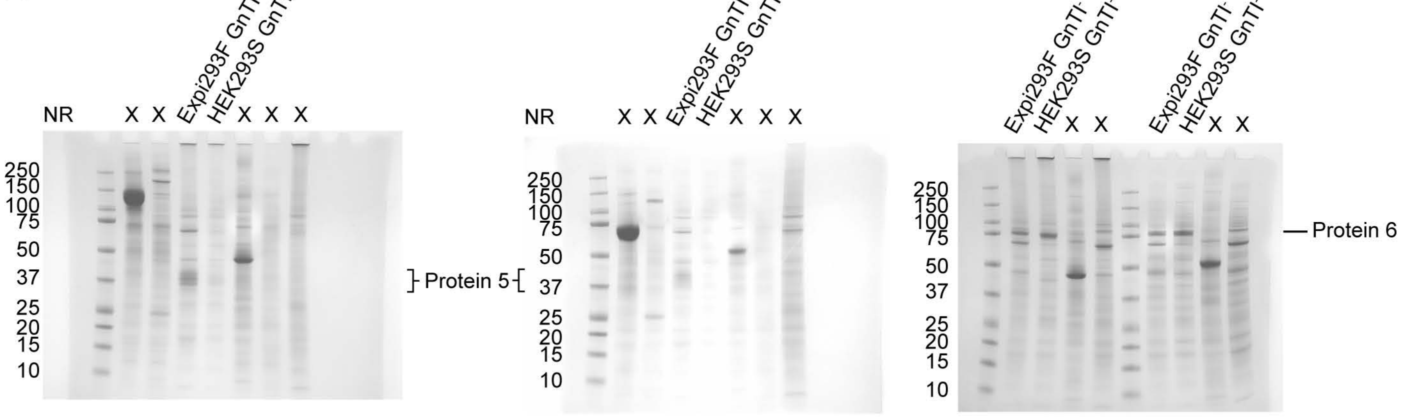

B, C

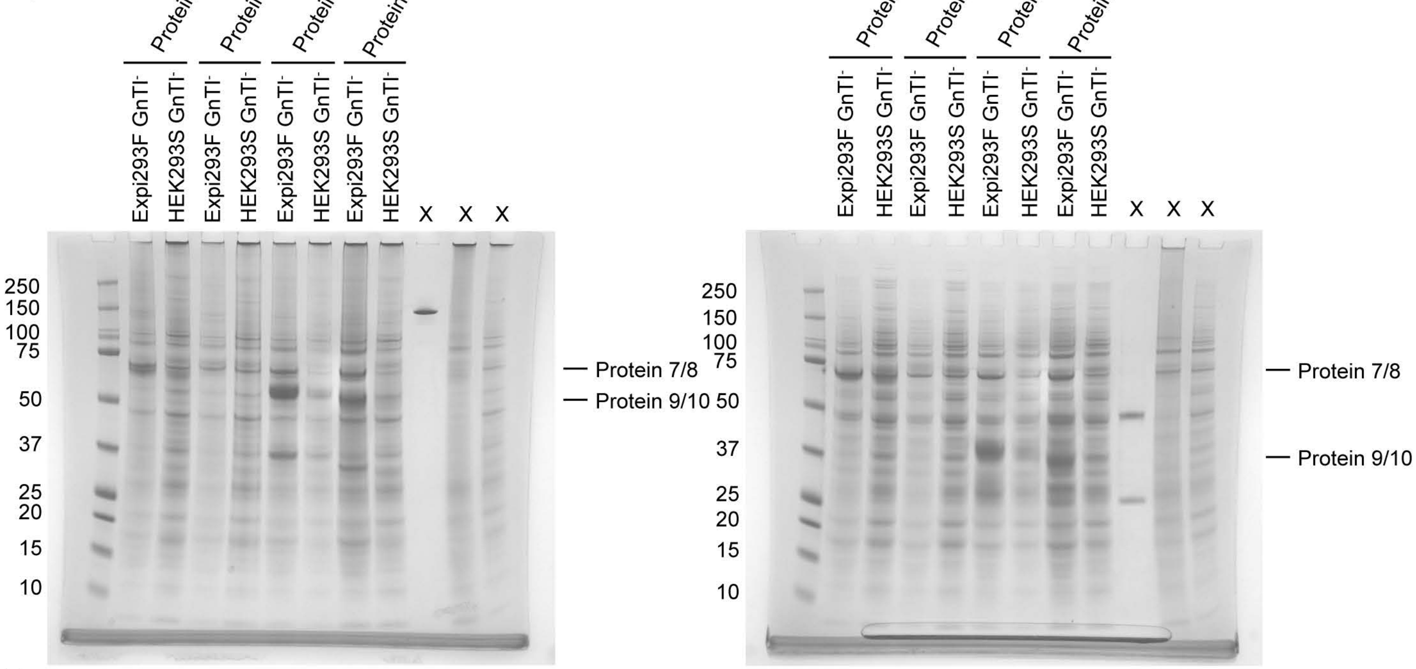

D

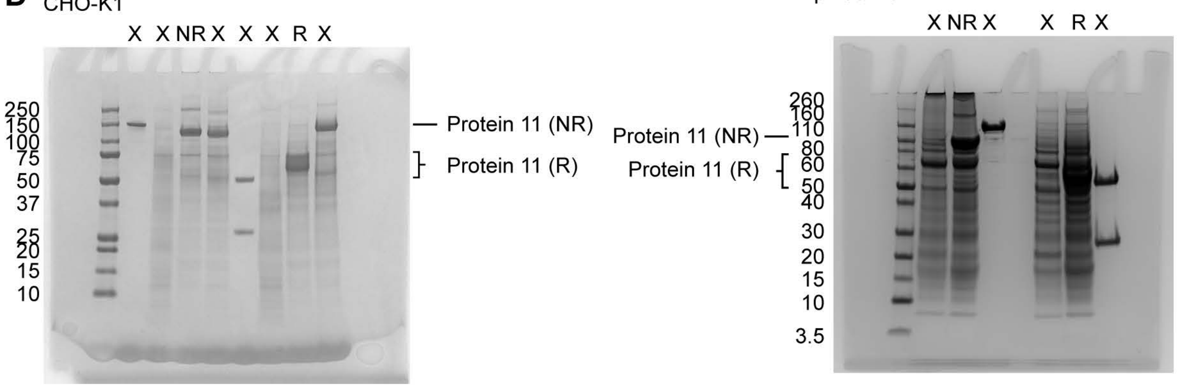

Supplement: S1 Raw images — (PDF) [file pone.0285971.s007.pdf]
